# Effects of Petroleum Ether Extract of *Amorphophallus paeoniifolius* Tuber on Central Nervous System in Mice

**DOI:** 10.4103/0250-474X.59547

**Published:** 2009

**Authors:** S. S. Das, Malini Sen, Y. N. Dey, S. De, A. K. Ghosh

**Affiliations:** Department of Pharmacology, Gupta College of Technological Sciences, Ashram More, Asansol-713 301, India

**Keywords:** *Amorphophallus paeoniifolius*, locomotor activity, muscle relaxant activity, central nervous system depressant activity

## Abstract

The central nervous system activity of the petroleum ether extract of *Amorphophallus paeoniifolius* tuber was examined in mice, fed normal as well as healthy conditions. The petroleum ether extract of *Amorphophallus paeoniifolius* tuber at the doses of 100, 300 and 1000 mg/kg showed significant central nervous system activity in mice.

A Central Nervous System activity refers to physiological depression of the central nervous system, general or local anesthesia, relaxation of skeletal muscles, or anticonvulsant activities. Many depressants and anesthetics acting on the central nervous system do so by increasing the activity of a particular neurotransmitter known as gamma-aminobutyric acid (GABA), although other targets such as the N-methyl D-aspartate (NMDA) receptor, μ opioid receptor and CB1 cannabinoid receptor can also be important, depending on which drug is involved. Skeletal muscle relaxants act peripherally at neuromuscular junction or in the cerebrospinal axis to reduce muscle tone[1].

In recent years the popularity of complementary medicine has increased. Over 50% of all modern drugs are natural product origin and they play an important role in drug development programs of the pharmaceutical industry[2]. Epidemiological evidence suggests that dietary factors play an important role in human health and in the treatment of certain chronic diseases including cancer[3,4]. Dietary measures and traditional plant therapies as prescribed by ayurvedic and other indigenous systems of medicine are used commonly in India[5]. The use of herbal medicines worldwide has provided an excellent opportunity to India to look for therapeutic lead compounds from our ancient system of therapy, i.e. Ayurveda, which can be utilized for development of new drug. *Amorphophallus paeoniifolius* known as Elephant foot yam is basically a crop of south East Asian origin. In India, it is commonly known as Suran or Jimmikand. It grows in wild form in Philippines, Malaysia, Indonesia and other South East Asian countries. This tuber is consumed by many people as a food and widely used in many Ayurvedic preparations[6].

The tubers of wild plants are highly acrid and cause irritation in throat and mouth due to excessive amount of calcium oxalate present in the tubers. The tubers are anodyne, antiinflammatory, antihaemorrhoidal, haemostatic, expectorant, carminative, digestive, appetizer, stomachic, anthelmintic, liver tonic, aphrodisiac, emmenagogue, rejuvenating and tonic. They are traditionally used in arthralgia, elephantiasis, tumors, inflammations, hemorrhoids, hemorrhages, vomiting, cough, bronchitis, asthma, anorexia, dyspepsia, flatulence, colic, constipation, helminthiasis hepatopathy, splenopathy, amenorrhea, dysmenorrhoea, seminal weakness, fatigue, anemia and general debility[7]. The tuber is reported to have antiprotease activity[8], analgesic activity[9], and cytotoxic activity[10].

To the best of our knowledge, the pharmacological properties of this tuber have not been studied extensively so far. Therefore, the aim of the present study was to determine the central nervous system activity of the petroleum ether extract of *Amorphophallus paeoniifolius* tuber in healthy mice.

## MATERIALS AND METHODS

*Amorphophallus paeoniifolius* (Araceae) tuber was purchased from local market of Asansol, West Bengal, India in September 2007. The tuber was identified by the Botanical Survey of India, Botanical Garden, Howrah with ref no. CNH/I-I/ (272)/ 2008/ Tech. II/ 314. Male Swiss albino mice (20-25 g) were obtained from animal house of Gupta College of Technological Sciences. The animals were housed under standard environmental condition (25°, 12 h light and 12 h dark cycle) and fed with standard diet (Tetragon Chemie Private Limited, Bangalore, India) and water ad libitum. The Animal Ethics Committee of Gupta College of Technological Sciences approved the experimental protocol [955/A/06/CPCSEA].

### Preparation of the extract:

The tuber of the plant was dried under shade and made to a fine powder using a laboratory mill and was extracted with petroleum ether (Merck, India) using a Soxhlet extractor. The isolated fraction was weighed and yield was calculated (yield=6.23%). Phytochemical screening of the petroleum ether extract indicated the presence of fat, steroids and fixed oil.

### Acute toxicity study:

An acute toxicity study was performed with the petroleum ether extract of the tuber using male Swiss albino mice. The method was performed as described by Miller and Tainter in 1944. The animals of male sex were divided into 5 groups each composed of 10 animals. The petroleum ether extract of *Amorphophallus paeoniifolius* was administered in the form of suspension in 5% v/v Tween 80 (Burgoyne Burbidges and company, Mumbai, India) as vehicle. All groups received intra-peritoneal injection (maximum 1 ml as per ethical norms).

Group I animals received petroleum ether extract at the dose of 1500 mg/kg, group II animals received petroleum ether extract at the dose of 2000 mg/kg, group III animals received petroleum ether extract at the dose of 2500 mg/kg, group IV animals received petroleum ether extract at the dose of 3000 mg/kg, group V animals received petroleum ether extract at the dose of 3500 mg/kg. After 24 h the mortality in each group was noted and mortality values were converted to probit values. The toxic dose was determined by plotting a graph between probit scale vs. log dose[11].

### Time course of central nervous system depression:

The time when the drug gives the maximum central nervous system depressant activity after the intraperitoneal administration is determined here. A dose of 300 mg/kg was selected and injected in the mouse through the intra-peritoneal route. The locomotor activity was noted. Similar response was taken for the other two doses. A mean value was obtained for the set of data. Finally a graph was plotted to find out the exact time. For the study of recovery time of CNS depressant activity, petroleum ether extract and diazepam were suspended in 5% v/v Tween 80 solution and given in the doses of 1000 mg/kg and 1.5 mg/kg, respectively. The control group received 5% v/v Tween 80 solution at the dose of 10 mg/kg.

### Evaluation of the central nervous system depressant activity:

The central nervous system activity was evaluated using an Actophotometer (Technoworld, Delhi, India) and Rota-Rod apparatus (Biological Museum, Agra, India). All the experiments were conducted on an isolated and noiseless condition. The experiments were performed with subject to minimum pain to the experimenting animals. All the ethical considerations have been followed. The petroleum ether extract of *Amorphophallus paeoniifolius* was administered in the form of suspension in 5% v/v Tween 80 (Burgoyne Burbidges and company, Mumbai, India) as vehicle. Diazepam injection (Ranbaxy, India) was bought and suspended in 5% v/v Tween 80 solution. The protocol is based as carried out by Turner. The animals of male sex were divided into six groups each composed of six animals. All groups received intra-peritoneal injection (maximum 1 ml as per ethical norms).

Group I control animals received 5% v/v Tween 80 at the dose of 10 ml/kg, group II animals received petroleum ether extract at the dose of 100 mg/kg, group III animals received petroleum ether extract at the dose of 300 mg/kg, group IV animals received petroleum ether extract at the dose of 1000 mg/kg, Group V animals received standard diazepam at the dose of 0.5 mg/kg, group VI animals received standard diazepam at the dose of 1.5 mg/kg. After 1 h of the administration of the drug, the animals were placed in an Actophotometer and Rota-Rod apparatus for 5 min and the locomotor activity and griping test was observed. The data was then compared with the control groups.

### Statistical Analysis:

Data obtained from pharmacological experiments was expressed as Mean±SD. Difference between the control and the treatments in these experiments were tested for significance using ANOVA followed by Dunnett's test. Values of P<0.05 were considered statistically significant.

## RESULTS AND DISCUSSION

**Fig. 1:**
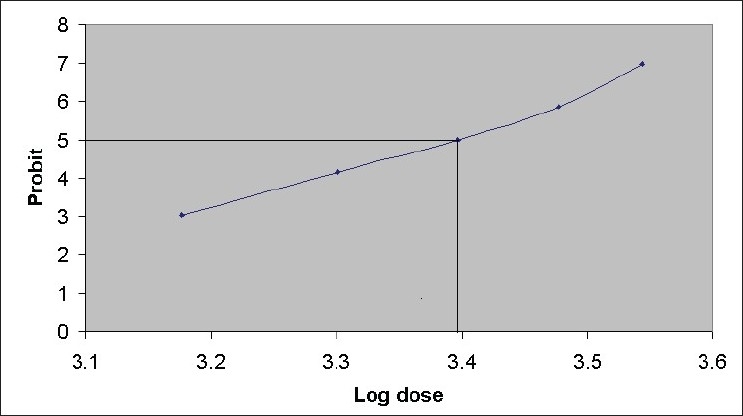
Determination of LD50 value of *Amorphophallus paeoniifolius*. All the mice were divided into 5 groups, each group received petroleum ether extract (1500, 2000, 2500, 3000, 3500 mg/kg respectively), suspended in 5% v/v Tween 80 solution. Mortality was noticed for 24 h and LD50 value was determined by using probit scale. n=10 for each group. The LD50 value was found out to be 2500 mg/kg body weight.

Acute toxicity studies showed mortality at 2500 mg/kg body weight (Table 1 and fig. 1), hence the extracts were regarded safe to be administered at lower doses. Acute toxicity studies indicate that petroleum ether extract of *Amorphophallus paeoniifolius* can be used safely up to doses of 1500 mg/kg body weight. A spontaneous dose-dependent decrease in central nervous system activity was found. The central nervous system depressants induce sedation and reduce the locomotor activity of the experimenting animal. The mechanism of this depression is not clearly understood at this point, but it can be assumed that the drug may exert central nervous system depressant effect by interfering with the function of the cortex. Neither the muscle relaxant activity has been understood.

**TABLE 1:** DATA OBTAINED FOR THE CALCULATION OF LETHAL DOSE (LD_50_ VALUE)

Group	Dose (mg/Kg)	Log dose	Dead/Total	% Dead	Corrected %	Probit
1	1500	3.176	0/10	0	2.5*	3.04
2	2000	3.301	2/10	20	20	4.16
3	2500	3.397	5/10	50	50	5.00
4	3000	3.477	8/10	80	80	5.84
5	3500	3.544	10/10	100	97.5*	6.96

* Corrected for 0 % dead = 100(0.25/n), and 100% dead = 100X (n-0.25/n)

**Fig. 2:**
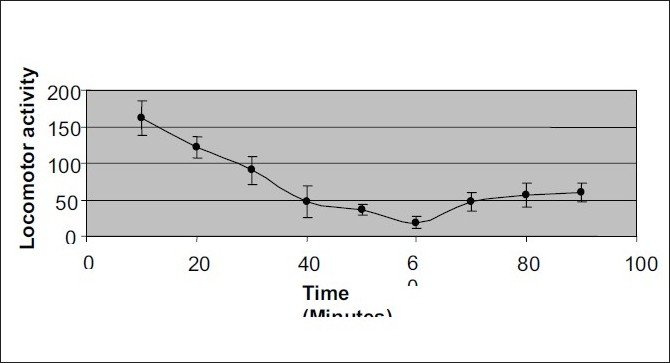
Time course curve of CNS depressant activity of *Amorphophallus paeoniifolius*. The mice were devided into 3 groups. Each group received petroleum ether extract suspended in 5% v/v Tween 80 solution at the dose of 100, 300, 1000 mg/kg through the intra-peritoneal route. Locomotor activity was measured by actophotometer at 10 min intervals for 100 min. The data is expressed as mean±SEM of n=6 observations.

**Fig. 3:**
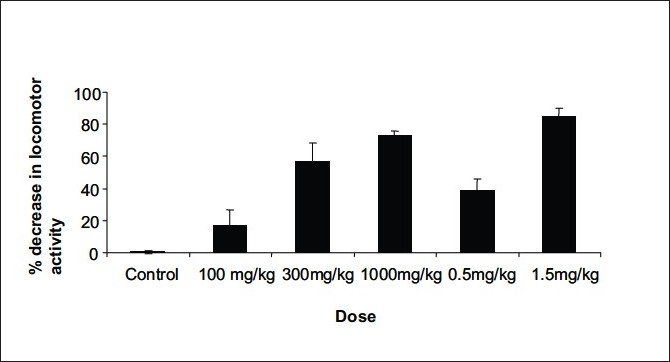
Dose dependent decrease of locomotor activity of mice. Tween 80 (10 mg/kg) as vehicle, petroleum ether extract of *Amorphophallus paeoniifolius* (100, 300, 1000 mg/kg) and diazepam (0.5, 1.5 mg/kg) as standard drug were administered intra-peritonially. After 60 min of drug treatment, decrease of locomotor activity was determined by actophotometer. The data is expressed as mean±SD of n=6 observations; *P<0.05 as compared to control; ANOVA followed by Dunnett's test. Control refers to 5% v/v Tween 80 solution.

**Fig. 4:**
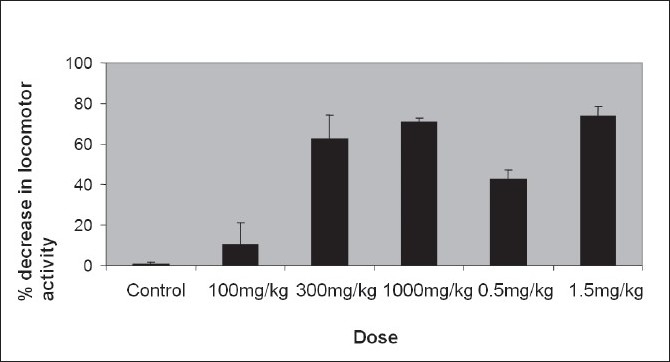
Dose response curve of *Amorphophallus paeoniifolius*. Tween 80 (10 mg/kg) as vehicle, petroleum ether extract of *Amorphophallus paeoniifolius* (100, 300, 1000 mg/kg) and diazepam (0.5, 1.5 mg/kg) as standard drug were administered intraperitonially. After 60 min of drug treatment, decrease of locomotor activity was determined by rota rod apparatus. The data is expressed as mean±SD of n=6 observations; *P< 0.05 as compared to control; ANOVA followed by Dunnett's test. Control refers to 5% v/v Tween 80 solution.

The peak central nervous system depression activity was observed at 60 min (fig. 2). The recovery time of CNS depressant activity of petroleum ether extract (1000 mg/kg) and diazepam (1.5 mg/kg) were found to be 24 h and 20 h, respectively (Table 2). The petroleum ether extracts of *Amorphophallus paeoniifolius* at the dose levels of 100, 300 and 1000 mg/kg body weight administered intraperitonially exhibited significant reduction in locomotor activity and in grip of the rotating rod, in a dose-dependent manner (Tables 3 and 4). The standard drug diazepam showed significant reduction in activity when compared with the control group of animals. Comparison of central nervous system activity of diazepam and petroleum ether extract of *Amorphophallus paeoniifolius* is shown in figs. 3 and 4.

**TABLE 2:** RECOVERY OF CNS DEPRESSANT ACTIVITY

Time(h)	Control group	Group receiving extract	Group receiving Diazepam
0	177.5±24.85	122.5±29.17	119.5±22.39
1	176 ± 23.35	25.25±3.77	22.75±3.30
4	-	32.25±10.07	30.5±4.86
8	-	52.5±14.52	46.25±9.81
12	-	65.25±10.62	66.25±10.01
16	-	79 ± 5.35	90.75±7.76
18	-	91±12.67	105±17.33
20	-	108±8.75	120±18.67
22	-	120±7.76	-
24	-	122.75±23.94	-

The recovery time of CNS depressant activity of mice after intraperitoneal injection of petroleum ether extract of *Amorphophallus paeoniifolius,* diazepam at the dose of 1000 mg/kg and 1.5 mg/kg respectively when measured in actophotometer. Control group received 5% v/v Tween 80 solution at the dose of 10 mg/kg. n=6, values are Mean±SD. The recovery time of extract and diazepam was found to be around 24 h and 20 h respectively.

**TABLE 3:** EFFECT OF THE PETROLEUM ETHER EXTRACT OF *AMORPHOPHALLUS PAEONIIFOLIUS* ON CNS

Group	Locomotor activity observed for 10 min		% Change in activity
		
	Before treatment	After treatment	
I	227.83±65.949	226.83±67.971	0.44
II	109.83±11.60	91.667±31.85*	16.53
III	198.16±47.363	85.66±62.637*	56.77
IV	140.33±27.148	33.16±5.149*	73.36
V	192.167±61.645	118±49.505*	38.59
VI	149±70.495	22.5±32.506*	84.89

n=6, values are mean±SD in each by using actophotometer. Comparison were made between group I Vs II, III, IV, V and VI, p value

* p>0.05.

**TABLE 4:** EFFECT OF THE PETROLEUM ETHER EXTRACT OF *AMORPHOPHALLUS PAEONIIFOLIUS* ON CNS

Group	Fall of time (s)		% Change in activity

	Before drug	After drug	
I	51.40±31.10	51.10±32.01	0.579
II	47.91±20.13	42.93±19.12*	10.38
III	28.61±11.45	10.68±4.74*	62.67
IV	39.18±13.10	11.44±3.93*	70.78
V	38.04±20.68	21.93±8.11*	42.32
VI	40.32±12.09	10.62±3.05*	73.66

n=6, Values are mean±SD in each by using rota rod apparatus. Comparison were made between group I Vs II, III, IV, V and VI, p value

* p>0.05.

Intra-peritoneal administration of vehicle (10 ml/kg) did not reduce locomotor activity significantly. After 60 min diazepam (0.5 mg/kg) did not reduce locomotor activity to a large extent (38.59% and 42.32%) but diazepam (1.5 mg/kg) decreased locomotor activity significantly (84.89% and 73.66%). Like diazepam, the intra-peritoneal administration of petroleum ether extract of *Amorphophallus paeoniifolius* tubers (100, 300, 1000 mg/kg) induced a significant decrease in locomotor activity and grip test in a dose-dependent manner. The percentage decrease in locomotor activity are 16.53 (P<0.05), 56.77 (P<0.05), 73.36 (P<0.05) (n=6) and percentage decrease in activity in grip test are 10.38 (P<0.05), 62.67 (P<0.05) and 70.78 (P<0.05) (n=6) 1 h after the intra-peritoneal administration of *Amorphophallus paeoniifolius* at the doses of 100, 300, 1000 mg/kg respectively. Our phytochemical screening shows the presence of steroids, in the petroleum ether extract of *Amorphophallus paeoniifolius* tubers. Further isolation of compounds and pharmacological investigation are underway to better characterize the active principle(s) and to evaluate mechanism on central nervous system activity.
